# Forest streams are important sources for nitrous oxide emissions

**DOI:** 10.1111/gcb.14812

**Published:** 2019-09-25

**Authors:** Joachim Audet, David Bastviken, Mirco Bundschuh, Ishi Buffam, Alexander Feckler, Leif Klemedtsson, Hjalmar Laudon, Stefan Löfgren, Sivakiruthika Natchimuthu, Mats Öquist, Mike Peacock, Marcus B. Wallin

**Affiliations:** ^1^ Department of Bioscience Aarhus University Silkeborg Denmark; ^2^ Department of Aquatic Sciences and Assessment Swedish University of Agricultural Sciences Uppsala Sweden; ^3^ Department of Thematic Studies – Environmental Change Linköping University Linköping Sweden; ^4^ Institute for Environmental Sciences University of Koblenz‐Landau Landau Germany; ^5^ Department of Biological Sciences University of Cincinnati Cincinnati OH USA; ^6^ Department of Earth Sciences University of Gothenburg Gothenburg Sweden; ^7^ Department of Forest Ecology and Management Swedish University of Agricultural Sciences Umeå Sweden; ^8^ Department of Earth Sciences, Air, Water and Landscape Sciences Uppsala University Uppsala Sweden

**Keywords:** agriculture, forest, greenhouse gas, nitrogen, nitrous oxide, river, stream

## Abstract

Streams and river networks are increasingly recognized as significant sources for the greenhouse gas nitrous oxide (N_2_O). N_2_O is a transformation product of nitrogenous compounds in soil, sediment and water. Agricultural areas are considered a particular hotspot for emissions because of the large input of nitrogen (N) fertilizers applied on arable land. However, there is little information on N_2_O emissions from forest streams although they constitute a major part of the total stream network globally. Here, we compiled N_2_O concentration data from low‐order streams (~1,000 observations from 172 stream sites) covering a large geographical gradient in Sweden from the temperate to the boreal zone and representing catchments with various degrees of agriculture and forest coverage. Our results showed that agricultural and forest streams had comparable N_2_O concentrations of 1.6 ± 2.1 and 1.3 ± 1.8 µg N/L, respectively (mean ± *SD*) despite higher total N (TN) concentrations in agricultural streams (1,520 ± 1,640 vs. 780 ± 600 µg N/L). Although clear patterns linking N_2_O concentrations and environmental variables were difficult to discern, the percent saturation of N_2_O in the streams was positively correlated with stream concentration of TN and negatively correlated with pH. We speculate that the apparent contradiction between lower TN concentration but similar N_2_O concentrations in forest streams than in agricultural streams is due to the low pH (<6) in forest soils and streams which affects denitrification and yields higher N_2_O emissions. An estimate of the N_2_O emission from low‐order streams at the national scale revealed that ~1.8 × 10^9^ g N_2_O‐N are emitted annually in Sweden, with forest streams contributing about 80% of the total stream emission. Hence, our results provide evidence that forest streams can act as substantial N_2_O sources in the landscape with 800 × 10^9^ g CO_2_‐eq emitted annually in Sweden, equivalent to 25% of the total N_2_O emissions from the Swedish agricultural sector.

## Graphical abstract

In this paper, we show that agricultural and forest streams in Sweden have comparable nitrous oxide (N_2_O) concentrations in spite of lower total nitrogen concentration in forest streams. We speculate that the low pH in forest streams and soils affects denitrification and yields higher N_2_O emissions. An estimate of the N_2_O emission from low‐order streams at the national scale revealed that ~1.8 × 109 g N_2_O‐N are emitted annually in Sweden with forest streams contributing about 80% of the total stream emission. Hence, our results provide evidence that forest streams can act as substantial N_2_O sources in the landscape.

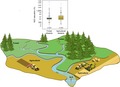

## 1 INTRODUCTION

Nitrous oxide (N_2_O) is a potent greenhouse gas with a global warming potential (GWP) about 300 times that of carbon dioxide (CO_2_) over a 100‐year timeframe (IPCC, 2013). N_2_O is also the current dominant ozone‐depleting substance, and N_2_O emissions thus have a negative impact on the recovery rate of the ozone hole (Ravishankara, Daniel, & Portmann, 2009). At a global scale, agriculture is the largest anthropogenic source of N_2_O, contributing 4.1 Tg N/year, that is, ~60% of all anthropogenic N_2_O emissions (Ciais et al., 2014).

Nitrous oxide is the result of biotic or abiotic transformations of nitrogenous compounds in soils, sediments or waters (Baggs & Philippot, 2011; Wrage, Velthof, van Beusichem, & Oenema, 2001), with nitrification and denitrification being two major processes. Nitrification is the microbial oxidation of ammonia (NH_3_) or ammonium (${\text{NH}}_{4}^{+}$) to nitrate (${\text{NO}}_{3}^{-}$); during the first step of this oxidation, namely the oxidation of NH_3_ or ${\text{NH}}_{4}^{+}$ into nitrite (${\text{NO}}_{2}^{-}$), N_2_O can be formed as an intermediate product (Prosser & Nicol, 2012). Denitrification is the sequential reduction of nitrogenous oxides (${\text{NO}}_{3}^{-}$ or ${\text{NO}}_{2}^{-}$) to gaseous forms (NO, N_2_O and N_2_; Tiedje, 1988; Wrage et al., 2001). The production of N_2_O is largely dependent on environmental conditions, and the major regulators are carbon and nitrogen (N) availability, temperature, pH and moisture (Mosier et al., 1998).

Soils and livestock management are the main anthropogenic sources of N_2_O in agricultural landscapes (Ciais et al., 2014). However, a fraction of N fertilizers applied onto fields can be leached to ground‐ and surface waters. During leaching and transport in ground‐ and surface waters, transformation processes (e.g., denitrification) result in the production of N_2_O, which is water‐soluble (Baggs & Philippot, 2011; Wrage et al., 2001). Hence, drainage networks (i.e., ditches and streams) are hotspots for N_2_O emissions (Reay et al., 2012; Rees et al., 2013). Studies on streams in the United States, France and Sweden have demonstrated that, although streams constitute only a small fraction of the total area in the landscape (~0.1%), they can have a disproportionately large impact on total N_2_O emissions from agriculture (3%–6%; Audet, Wallin, Kyllmar, Andersson, & Bishop, 2017; Beaulieu, Arango, Hamilton, & Tank, 2008; Grossel et al., 2016). Considering that the consumption and use of agricultural N fertilizer is increasing to meet the food demand of the growing global population (Bodirsky et al., 2014), it is likely that agricultural N_2_O emissions will continue to increase in the future and contribute to climate forcing and ozone depletion (Ravishankara et al., 2009; Reay et al., 2012).

Consequently, many N_2_O studies have focused on streams draining agricultural areas, resulting in a lack of data on N_2_O emissions from streams within other types of land use, especially forest streams (Davidson & Swank, 1990; Holl, Jungkunst, Fiedler, & Stahr, 2005; Vidon & Serchan, 2016), despite the potential of forested catchments to process and transform N (e.g., Brookshire, Valett, Thomas, & Webster, 2005; Kortelainen et al., 2006; Sponseller et al., 2016). Estimation of N_2_O emissions from forest streams would be especially relevant in countries where forest covers large proportions of the total land mass such as Finland (73%), Sweden (69%), Russia (50%) and Canada (38%; FAO, 2015). Hence, even if it is likely that forest streams have much lower N availability and less N_2_O emissions per unit area than agricultural streams, the former might still be a larger N_2_O source at the national and global scale. Such information is crucial for developing targeted and effective mitigation schemes aiming at reducing N_2_O emissions.

To fill the knowledge gap, we assembled a unique data set comprising approximately 1,000 stream N_2_O concentration measurements from agricultural and forest streams in Sweden. We focused especially on low‐order streams (Strahler order ≤ 4) because of their strong hydrological and hydrochemical connectivity with surrounding soils and the fact that they often constitute the majority of the total stream length (Bishop et al., 2008). We hypothesized that (a) streams in forested catchments will have lower N_2_O concentrations than streams draining agricultural catchments because of lower N availability; (b) when scaled to the national level, Swedish forest streams will emit more N_2_O than agricultural streams due to their greater length and surface area.

## 2 MATERIAL AND METHODS

### 2.1 Data set and site descriptions

**Figure 1:**
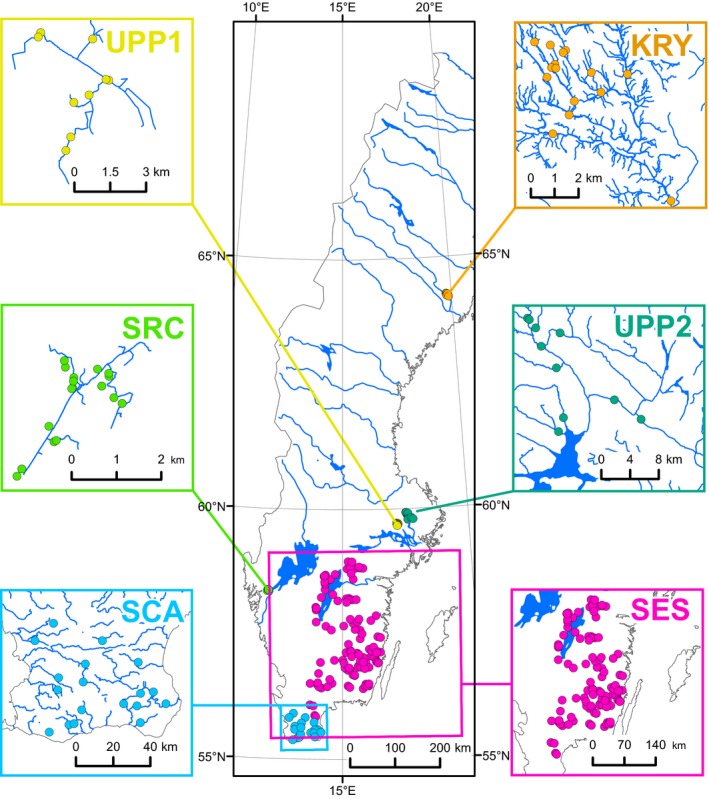
Map of Sweden showing the locations of the different regions/catchments where N_2_O samples were collected. The spatial distribution of sampling sites within each region or catchment is shown in the respective inserts. KRY, Krycklan; SCA, Scania; SES, South‐East Sweden; SRC, Skogaryd Research Catchment; UPP, Uppsala

The data set of the present study comprises direct concentration measurements of N_2_O from Swedish streams. The data set is a combination of catchment and regional surveys performed during 2004–2017 in six catchments or regions: Krycklan (KRY), South‐East Sweden (SES), Skogaryd Research Catchment (SRC), Scania (SCA), and Uppsala 1 and 2 (UPP1 and UPP2). The sites spanned a large geographical range of Sweden from approximately 55°N to 64°N, thereby covering most climatic zones with the exception of the sub‐Arctic (Figure 1). All data were collected from low‐order streams (Strahler order ≤ 4), except for two sampling sites at UPP2 where the Strahler order was 5.

The mean annual precipitation and temperature at the sites ranged from 550 to 900 mm and from 2 to 7°C, respectively (Table 1). The area of the subcatchments at the sampling sites ranged from 0.03 to 834 km^2^. The catchments at KRY, SES and SRC were dominated by forest land use (average >80%), while the streams at SCA, UPP1 and UPP2 had an average of 69%, 49% and 36% of agricultural land use in their respective catchments. Wetlands were also present at some of the sites, especially at KRY (mean cover 17%; Table 1).

**Table 1:** Characteristics and sampling information on the streams sampled in six regions or catchments

	KRY	SES	SRC	SCA	UPP1	UPP2
Latitude	64°N	56–59°N	58°N	55°N	59°N	58°N
Longitude	19°E	14–16°E	13°E	12–14°E	17°E	12°E
Mean annual temperature (°C)	2	5–7	7	7	6	5
Precipitation (mm/year)	630	450–600	900	600–700	550	600
Subcatchment area (km^2^)	0.03–68	0.9–7.3	0.3–7	9–118	0.5–32	9–834
Strahler stream order	1–4	1	1–2	1–3	1–3	1–5
Total number of observations	420	227	41	72	130	96
Number of sampled sites	15	103	17	18	9	10
Year of sampling	2004	2016–2017	2014	2016	2016–2017	2017
Month of sampling (month number)	1–12	3, 4, 8, 9, 11, 12	3, 4, 7	5, 6	1–12	6–11
N_2_O (µg N/L)	1.3 (0.4–19.6)	1.6 (0.2–28.8)	0.8 (0.5–1.1)	0.9 (0.4–3.5)	2.0 (0.3–15.7)	1.4 (0.5–15.3)
N_2_O saturation (%)	269 (114–3,630)	370 (48–6,650)	221 (123–355)	297 (140–1,040)	452 (77–4,700)	380 (146–3,400)
TN (µg N/L)	410 (150–1,280)	1,020 (220–5,150)	690 (430–1,050)	3,300 (920–13,100)	1,620 (5–9,200)	1,050 (5–6,460)
DOC (mg/L)	17.5 (3.6–46.2)	27.8 (2.5–150)	25.5 (19.3–51.2)	8.1 (4.1–15.4)	8.6 (2.6–20.5)	12.4 (4.1–48.1)
pH	5.4 (3.9–7.0)	5.4 (4.0–8.1)	6.1 (4.8–6.9)	8.0 (7.3–8.6)	7.8 (6.8–9.3)	7.8 (7.4–8.4)
Land use (%)						
Agriculture	0.6 (0–4)	1 (0–5)	4 (0–10)	67 (9–97)	49 (0–63)	36 (23–79)
Forest	82 (59–100)	82 (48–100)	85 (70–100)	20 (0–74)	42 (25–84)	55 (8–93)
Wetland	17 (0–40)	3 (0–44)	2 (0–4)	1 (0–7)	3 (0–16)	1 (0–2)

Note

Mean values and range (in parentheses) of measured variables are shown.

Abbreviations: DOC, dissolved organic carbon; KRY, Krycklan; SCA, Scania; SES, South‐East Sweden; SRC, Skogaryd Research Catchment; TN, total nitrogen; UPP, Uppsala.

The KRY data were collected between January and December 2004 (~28 sample collections) at 15 stream sampling sites within the boreal KRY catchment as part of the Krycklan Catchment Study (Laudon et al., 2013). The sites at SES represent first‐order streams that were part of a seasonal survey in late summer and autumn 2016 as well as spring 2017 (Hawkes et al., 2018; Wallin et al., 2018). Approximately 100 sites were included in each seasonal survey, except in summer 2016 when only 38 sites were sampled due to drought. SRC consisted of 17 sampling sites that were visited two to four times between March and July 2014 (see Natchimuthu, Wallin, Klemedtsson, & Bastviken 2017) for more information on the catchment). The sites at SCA in southern Sweden comprised 18 streams that were visited four times in May–June 2016. The catchment sampled at UPP1 is part of the Swedish national monitoring program for agriculture (catchment C6; Kyllmar, Carlsson, Gustafson, Ulen, & Johnsson, 2006; Kyllmar, Forsberg, Andersson, & Martensson, 2014). Monthly measurements of N_2_O were performed from August 2016 to November 2017 at nine stream sites draining primarily agriculture‐dominated subcatchments. Further details on the UPP1 stream sites are available in Audet et al. (2017). The 10 sites at UPP2 were visited 3–11 times between June and November 2017. Some of the streams dried out during the summer drought of 2017 and could not be sampled on every visit. For more information on the sampling in each catchment or region, see Table 1.

#### 2.1.1 Water chemistry

Grab samples of stream water were taken for nutrient analysis at all sites at every visit. Stream water pH was recorded at the majority of the sites. At KRY, pH was measured at room temperature after returning to the laboratory using a Ross 8102 low‐conductivity combination electrode (ThermoOrion; Buffam, Laudon, Temnerud, Mörth, & Bishop, 2007). At SCA, pH was measured directly in the field using a WTW ProfiLine Multi 3320. At SES, pH was measured at room temperature upon arrival at the laboratory using a titrosampler Metrohm 855 with a built‐in pH probe. At SRC, pH was measured in situ using a Hach HQ40D‐PHC10105 pH electrode at eight of the 16 sampling sites. At UPP1‐2, pH was measured directly in the field using a Multiparameter Meter Hi 9829 from Hannah Instruments. Total organic carbon (TOC) was measured in the water samples from KRY, SCA and SES, while dissolved organic carbon (DOC) was determined at SRC and UPP1‐2. TOC is generally equivalent to DOC in Swedish forest streams (Laudon et al., 2011; Laudon, Köhler, & Buffam, 2004) and will, therefore, hereafter be referred to as DOC. Total N (TN) was measured in samples from KRY, SCA, SES and SRC, whereas only ${\text{NO}}_{3}^{-}$ was measured at UPP1‐2. However, the ${\text{NO}}_{3}^{-}$ fraction generally constitutes most of TN in sites dominated by agricultural land use (Kyllmar et al., 2014) and will, therefore, for simplicity, be referred to as TN hereafter. All chemical analyses were performed according to Swedish standard methods (Fölster, Johnson, Futter, & Wilander, 2014). Stream water temperature was recorded upon sampling at all sites except at SRC where the temperature was recorded at the most downstream sampling site in the catchment.

#### 2.1.2 In‐stream concentrations of N_2_O

The data set of in‐stream concentrations of N_2_O was formed by combining results from several sampling campaigns which used different protocols but all relied on headspace equilibration method (McAuliffe, 1971) and gas chromatography (GC) analyses. At KRY, water samples were collected in N_2_‐filled 60 ml glass vials sealed with a bromobutyl rubber septa. For each sample, a 15 ml aliquot of bubble‐free water was injected into the glass vial, subsequently acidified to pH 2–3 with one drop of 30% ultrapure HCl (0.5% v/v) and stored cold at ~2°C. At SCA, SES, UPP1 and UPP2, 10 ml of stream water was collected in a 22.5 ml gas‐tight glass vial preflushed with N_2_; the vials also contained 0.2 ml of ZnCl 50% (w:v) for sample preservation. At SRC, 5 ml stream water was added to 20 ml vials preflushed with N_2_ and containing 100 µl H_3_PO_4_ for sample preservation. The samples were stored in the dark until analysis generally within a week and up to a maximum of 1 month. The headspace N_2_O concentrations in the vials from all sites were directly analyzed by GC with electron capture detector (GC‐ECD). The GC brands varied among laboratories, but certified N_2_O standards were used in all cases for calibration and validation.

Headspace N_2_O concentrations obtained after GC analysis were converted into dissolved N_2_O concentrations (*C*_obs_ ) using the N_2_O solubility function by Weiss and Price (1980) and taking into account the stream water temperature and atmospheric pressure at the sampling time. Data on atmospheric pressure were obtained from the closest monitoring station from the Swedish Meteorological and Hydrological Institute. Given that N_2_O dissolution is temperature dependent, we calculated the percent saturation (%sat) to facilitate comparisons between sites and seasons:

(1) $$\%\text{sat}=(C_{\text{obs}}/C_{\text{eq}})\times 100,$$

where *C*_eq_ is the concentration of N_2_O if the stream water was in equilibrium with the atmosphere, assuming an atmospheric partial pressure of 330 ppb for N_2_O. Percent saturation >100 indicates supersaturation of the stream water and thus emission of N_2_O to the atmosphere.

#### 2.1.3 Estimate of total N_2_O emissions from low‐order streams in Sweden

The total N_2_O emissions from low‐order streams in Sweden were estimated using the same approach as in Wallin et al. (2018), where a national estimate of CO_2_ and CH_4_ emissions from low‐order streams was derived. Wallin et al. (2018) provided estimates of gas transfer velocities for CO_2_ (*k*_600_) for every combination of stream order (1–4) and land‐use class (i.e., agriculture or forest). The gas transfer velocities were modeled based on slope, catchment area and daily specific discharge for more than 400,000 stream segments. The mean values of gas transfer velocities for CO_2_ (*k*_600_) specified in Wallin et al. (2018) were converted to $k_{N_{2}O}$ following Wanninkhof (1992):

$$k_{N_{2}O}=k_{600}{\left(\frac{Sc_{N_{2}O}}{600}\right)}^{-0.5},$$

where $Sc_{N_{2}O}$ is the Schmidt number calculated as described in Wanninkhof (1992), accounting for changes in water temperature. In‐stream N_2_O concentrations from UPP1, UPP2 and SCA were selected to represent agricultural stream concentrations, whereas the N_2_O concentrations from KRY, SES and SRC represented forest stream concentrations. The annual emission of N_2_O (g/year) $E_{N_{2}O}$ was calculated for each combination of stream order and land‐use class using corresponding values of $\Delta_{N_{2}O}$, $k_{N_{2}O}$ and *A*_S_ (see Table 2) as follows:

$$E_{N_{2}O}=365\times \Delta_{N_{2}O}\times \bar{k_{N_{2}O}}\times A_{S},$$

where $\Delta_{N_{2}O}$ (mg N/L) is the mean difference between the in‐stream N_2_O concentration and the concentration that would be present in the water if the stream was in equilibrium with the atmosphere, assuming an atmospheric concentration of 330 ppb; *A*_S_ is the stream surface area (m^2^) and $\bar{k_{N_{2}O}}$ is the average daily stream gas transfer velocity of N_2_O (m/day). The national estimate of N_2_O emissions was obtained by summing the emission from all stream order and land‐use combinations. Due to lack of stream N_2_O concentration data for alpine regions, which represent only 6.5% of the total stream surface area in Sweden, these were not included in our assessment.

**Table 2:** Parameters used in the national estimate of N_2_O emission from Swedish streams and results

Stream order	Stream width (m)	Total stream network^a^		Stream surface per land use class^a^		Gas transfer velocities *k*_600_			Stream N_2_O emissions	
						Mean [median (10th−90th percentiles)]^a^			Mean [10th−90th percentiles]	
		Length (km)	Surface area (km^2^)	Forest (km^2^)	Agricultural (km^2^)	Total network (m/day)	Forest (m/day)	Agricultural (m/day)	Forest (10^6^g N_2_O‐N/year)	Agricultural (10^6^g N_2_O‐N/year)
1	0.7	228,993	164	125	26	11.0 [4.1 (0.2–24.1)]	9.3 [4.5 (0.3–21.7)]	3.7 [1.7 (0.1–8.3)]	441 [402–476]	46 [40–51]
2	1.6	101,521	165	122	31	10.7 [4.7 (0.4–23.7)]	9.8 [5.3 (0.4–22.1)]	4.4 [2.4 (0.2–9.6)]	354 [317–378]	48 [43–54]
3	3.7	47,650	175	126	39	10.6 [5.3 (0.5–24.2)]	10.5 [6.0 (0.6–23.9)]	5.3 [3.3 (0.4–12.1)]	337 [298–352]	95 [72–116]
4	8.4	23,244	194	138	48	9.7 [5.6 (0.6–22.3)]	10.3 [6.4 (0.6–23.2)]	6.1 [3.7 (0.5–13.9)]	271 [262–279]	191 [113–237]
Sum		401,410	697	511	144				1,404 [1,347–1,456]	380 [299–435]

a From Wallin et al. (2018).

### 2.2 Statistics

The statistical analyses were performed using the open source statistical software R version 3.4.4 for Windows (R Development Core Team, 2018), with the package ‘nlme’ and the function ‘lme’ therein (Pinheiro, Bates, DebRoy, Sarkar, & R Development Core Team, 2012). Linear mixed effect models were used to explore linkages between N_2_O %sat and selected environmental variables, as these models are particularly suitable to examine the patterns in time series datasets from different sites (Zuur, Ieno, Walker, Saveliev, & Smith, 2009). The mixed models were checked for normality and homogeneity of variance by visual inspection of plots of residuals against fitted values (Zuur et al., 2009). The significance of the models was assessed by comparison with a null‐model using the likelihood ratio. The potential predictor variables were checked for multicollinearity using the variance inflation factor (VIF) values (VIF < 10 indicating low risk of multicollinearity). We used spatial correlograms (function spline.correlog in the R package ‘ncf’; Bjornstad, 2018) to verify the absence of spatial autocorrelation in the residuals of the models. Finally, we tested the presence of temporal autocorrelation in the mixed models by adding the correlation structure ‘corAR1’ from the package ‘nlme’ and examining the residuals (Pinheiro & Bates, 2000; Pinheiro et al., 2012). All N_2_O observations and corresponding ancillary variables were included in the following models.

The aim of the first analysis was to test whether N_2_O %sat and potential regulators of N_2_O production (TN, pH and DOC) differ between forest and agricultural streams. Hence, N_2_O %sat, TN, pH and DOC were individually tested for significant differences between forest (KRY, SES and SRC) and agricultural (UPP1, UPP2 and SCA) streams (Table S1, models 1–4). To reduce variance heterogeneity in the data and to meet the assumptions of linear mixed effect models, N_2_O %sat was transformed using natural logarithm before inclusion in the models. The regions or catchments were added as a random effect. Using the same approach, the differences in N_2_O %sat in forest and agricultural streams across seasons and stream order were also tested (Table S1, models 5–8). When season or stream order was found significant in the models, the variations among the different seasons or stream orders were tested using Tukey's posthoc test. In a second analysis, the aim was to test the effect of selected potential regulators of N_2_O %sat. Only continuous variables (i.e., noncategorical) were included in this analysis because the goal was to develop a general model of stream N_2_O %sat. TN, DOC, pH, percentage agricultural land in the subcatchments, percentage wetland in the subcatchments and water temperature were added as fixed effects in the models; the regions or catchments were added as random effects (Table S1, model 9).

We used Monte Carlo simulations (mean of 10,000 repetitions of a Monte Carlo simulation with 10,000 iterations) in R to estimate the uncertainty of the total N_2_O emissions from low‐order streams. The level for significance of all analyses was set at *p* < .05.

## 3 RESULTS

**Figure 2:**
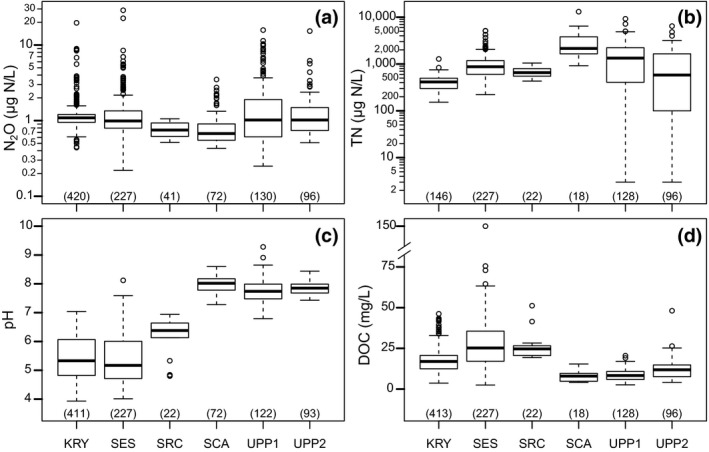
Boxplots of (a) dissolved N_2_O concentrations, (b) TN concentrations, (c) pH and (d) DOC concentrations in stream water grouped by regions/catchments. Forested sites: KRY, SES and SRC; agricultural sites; SCA, UPP1, UPP2. The number in parentheses on the *x*‐axis in each boxplot indicates the number of samples. DOC, dissolved organic carbon; KRY, Krycklan; SCA, Scania; SES, South‐East Sweden; SRC, Skogaryd Research Catchment; TN, total nitrogen; UPP, Uppsala

The mean (±*SD*) stream N_2_O concentration across all sites was 1.4 ± 1.9 µg N/L (median 1.0 µg N/L). In general, the N_2_O concentration within a single region or catchment was variable both in time and space and comparable with the variation across catchments/regions, except at SRC where the N_2_O concentrations were less variable (Figure 2a). All streams were almost always supersaturated in N_2_O (99% of the samples), meaning that they acted as sources of N_2_O to the atmosphere (Table 1). Only 10 samples (six at UPP1 and four at SES) were undersaturated (mean 85%sat N_2_O). Total N varied greatly across the sites, and higher values were observed in the agricultural catchments SCA (3,300 ± 2,940 µg N/L), UPP1 (1,620 ± 1,490 µg N/L) and UPP2 (1,050 ± 1,200 µg N/L) than at the rest of the sites, although SES also had a few higher values (1,020 ± 680 µg N/L; Figure 2b; Table 1). Stream water pH was also higher in the agricultural catchments SCA (8.0 ± 0.3), UPP1 (7.8 ± 0.4) and UPP2 (7.8 ± 0.2) compared with KRY (5.4 ± 0.8), SES (5.4 ± 0.9) and SRC (6.1 ± 0.7; Figure 2c; Table 1). DOC was generally higher in the forested catchments KRY, SES and SRC (17.5 ± 7.1, 27.8 ± 16.0 and 25.5 ± 7.4 mg/L, respectively) than that at SCA and UPP1‐2 (8.6 ± 3.6, 12.4 ± 3.4 and 8.1 ± 6.5 mg/L, respectively; Figure 2d; Table 1).

**Figure 3:**
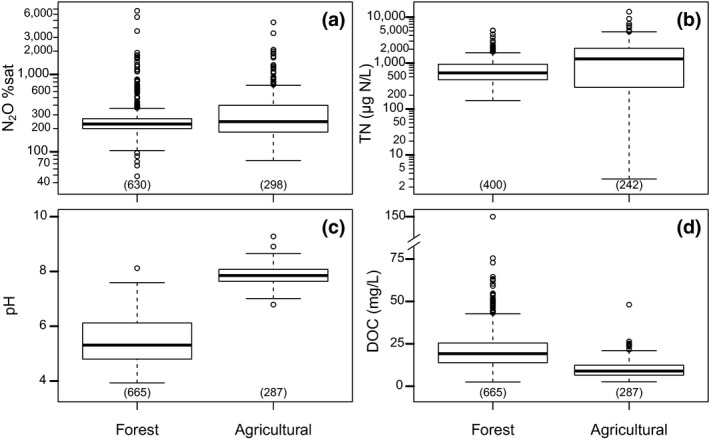
Boxplots of (a) dissolved N_2_O %sat, (b) TN concentrations, (c) pH and (d) DOC concentrations in stream water grouped by regions/catchments. The number in parentheses on the *x*‐axis in each boxplot indicates the number of samples. DOC, dissolved organic carbon

**Figure 4:**
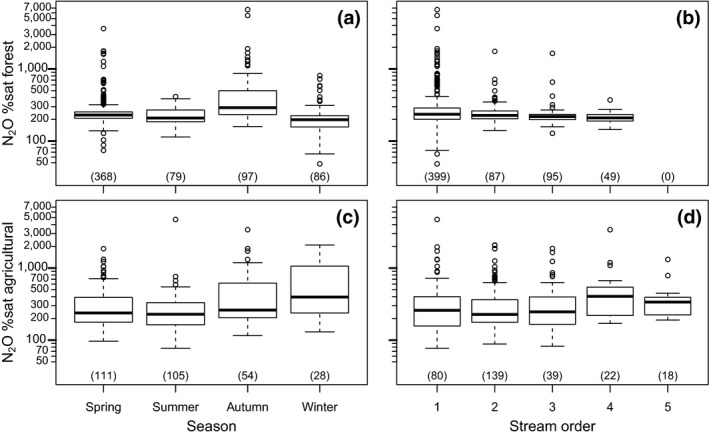
Percent of N_2_O %sat in forest stream water (a, b) or agricultural stream water (c, d) grouped by season and stream order. The number in parentheses on the *x*‐axis in each boxplot indicates the number of samples

The different regions or catchments were grouped into two categories based on their land use, that is, agricultural (UPP1, UPP2, SCA) or forest (KRY, SES, SRC). The N_2_O %sat did not differ significantly (*p* = .14) between agricultural and forest land use (392 ± 462% and 302 ± 412%, respectively; Figure 3a; Table S1). Total N concentration and pH were significantly higher (*p* < .049 and .001) in agricultural than in forest streams (TN 1,520 ± 1,640 and 780 ± 600 µg N/L, and pH 7.8 ± 0.3 and 5.4 ± 0.8, respectively; Figure 3b,c; Table S1). DOC was significantly higher (*p* = .008) in the forest than in the agricultural streams (21 ± 13 and 10 ± 5 mg/L, respectively; Figure 3d; Table S1). Dissolved N_2_O %sat in forest streams varied seasonally (Figure 4a), with significantly greater mean values in autumn than in spring, summer and winter (546 ± 880, 273 ± 254, 227 ± 68, 221 ± 124%; Table S2). Dissolved N_2_O %sat in forest streams seemed to decrease with increasing stream order (Figure 4b). Dissolved N_2_O %sat was significantly greater in first‐order streams (336 ± 502%) compared with third‐ (238 ± 157%) and fourth‐order streams (215 ± 37%; Table S2). Furthermore, second‐order forest streams (269 ± 184%) also showed significantly higher N_2_O %sat than fourth‐order streams (Table S2). Dissolved N_2_O %sat in agricultural streams (Figure 4c) was significantly greater in winter (721 ± 655%) compared with spring (284 ± 343%) and summer (302 ± 455%; Table S2). Fourth‐order agricultural streams (570 ± 689) had significantly greater N_2_O %sat than second‐ (347 ± 346) and third‐order streams (392 ± 405; Figure 4d; Table S2).

The observation of the plots of N_2_O %sat against percentage of agricultural land, TN, pH and DOC (Figure S1) suggested that greater TN is associated with greater N_2_O %sat, while no clear pattern emerged from the other plots. The mixed models revealed that N_2_O %sat was positively correlated with TN concentration and negatively correlated with pH (Table 3). According to the mixed models, DOC, the percentage agricultural land, the percentage of wetlands in the catchment and water temperature did not have a significant effect on N_2_O %sat (Table 3).

**Table 3:** Results from the linear mixed models testing the effect of selected environmental variables on the percentage saturation of N_2_O (ln‐transformed values)

Parameter estimates	Value	*SE*	95% CI	*n*	*df*	*t*‐Value	*p*‐Value
Intercept	6.44	0.24	5.97–6.92	623	611	26.4	**<.001**
TN (µg N/L)	0.00023	0.00003	0.00018–0.00028	623	611	8.9	**<.001**
pH	−0.17	0.03	−0.23 to −0.11	623	611	−5.2	**<.001**
Agricultural land (%)	0.003	0.002	−0.001 to 0.006	623	611	1.4	.16
Wetland (%)	−0.0005	0.002	−0.005 to 0.004	623	611	−0.2	.82
DOC (mg/L)	−0.003	0.002	−0.008 to −0.015	623	611	−1.3	.20
Water temperature (°C)	−0.0001	0.005	−0.011 to –0.010	623	611	−0.02	.98

Note

Bold *p*‐values indicate statistical significance.

Abbreviations: CI, confidence interval; *df*, degree of freedom; DOC, dissolved organic carbon; *n*, number of observations; TN, total nitrogen.

The national estimate of total N_2_O emissions from Swedish low‐order streams amounted to 1,780 × 10^6^ g N_2_O‐N/year (10th–90th percentile: 1690–1870 × 10^6^ g N_2_O‐N/year; Table 2). Converted into CO_2_‐equivalent using a GWP of 298, the N_2_O emissions from streams represent about 830 × 10^9^ g CO_2_‐eq/year (10th–90th percentile: 790–880 × 10^9^ g N_2_O‐N/year). The contribution from forest streams constituted about 80% of the total N_2_O emissions from streams. The total N_2_O emissions from both forest and agricultural streams corresponded well with their areal coverage in the landscape (Table 2).

## 4 DISCUSSION

The results of the present study reveal that Swedish streams are sources of N_2_O to the atmosphere, both in forested and agricultural catchments. The N_2_O concentrations reported here (median 1.0 µg N/L; range 0.2–28.8 µg N/L) were comparable with values previously published for streams in the temperate zone (e.g., Beaulieu et al., 2008; Davis & David, 2018; Peacock, Ridley, Evans, & Gauci, 2017; Reay, Edwards, & Smith, 2009). However, previous studies have primarily focused on agricultural areas, largely ignoring forest streams. Most of the few available studies of forest streams showed low N_2_O concentrations (mean < 0.6 µg N/L; Davidson & Swank, 1990; Vidon & Serchan, 2016), although the median N_2_O concentration (1.1 µg N/L) found in a forest stream in Germany (Holl et al., 2005) was close to our results.

Contrary to our first hypothesis, N_2_O concentrations in forest streams were relatively similar to concentrations recorded in agricultural streams, although TN concentrations were higher in the latter. Still, results indicated that an increase in TN appeared to increase the N_2_O concentration, thus confirming the relationship linking N availability and N_2_O concentration as well as emission observed in previous studies (Beaulieu et al., 2011; Reay, Smith, & Edwards, 2003; Turner et al., 2016). However, other factors also likely influenced N_2_O concentrations irrespective of land use. For instance, it appeared in our study that low pH was linked to higher N_2_O concentrations. Several studies have demonstrated a shift in the molar ratio of N_2_O:(N_2_O + N_2_) during denitrification when pH decreases (Bergaust, Mao, Bakken, & Frostegård, 2010; Liu, Mørkved, Frostegård, & Bakken, 2010; Nömmik, 1956). Low pH suppresses N_2_O‐reductase activity, partially inhibiting reduction of N_2_O to N_2_ (Bakken, Bergaust, Liu, & Frostegård, 2012; Liu et al., 2010; Stevens, Laughlin, & Malone, 1998). For example, in a laboratory experiment it was shown that the proportion of N_2_O produced as the terminal product of denitrification was 79% in acidic soil slurries (pH~5.5), while it was only 10% in alkaline soil slurries (pH~7.6; Čuhel et al., 2010). Coniferous and boreal forest soils and streams generally have a low pH (<6; Figure 3c) and the suppression of N_2_O‐reductase activity due to low pH might explain why forest streams have relatively high N_2_O concentrations despite significantly lower TN concentrations. If this conjecture is true, this would mean that the ratio N_2_O:${\text{NO}}_{3}^{-}$ was higher at the forest streams than at the agricultural streams. Nitrate was analyzed only at one of the forest regions (SES) in our data set where it constituted about 18% of TN. This proportion is in reasonable agreement with previous research at KRY and other boreal catchments (Kortelainen et al., 2006; Sponseller, Blackburn, Nilsson, & Laudon, 2018) and if we consider that the same proportion holds true at the other forest regions, this would confirm that the ratio N_2_O:${\text{NO}}_{3}^{-}$ is higher at the forest streams than at the agricultural streams (0.010 [0.004–0.023] and 0.0013 [0.0003–0.019], respectively; median [10th–90th percentile]). The potential influence of pH on N_2_O emissions might be especially important in Swedish soils that have been subjected to acid deposition (Eriksson, Karltun, & Lundmark, 1992). The current recovery from acidification observed in many streams in Northern Europe and North America (Garmo et al., 2014; Kothawala, Watmough, Futter, Zhang, & Dillon, 2011) opens the question of whether stream N_2_O emissions from acidified forested areas are experiencing a decreasing trend.

Another plausible explanation for the relatively high N_2_O concentration in forest streams could be that N_2_O is produced by chemodenitrification, which is the abiotic reduction of oxidized N species (i.e., ${\text{NO}}_{2}^{-}$ and ${\text{NO}}_{3}^{-}$) by ferrous iron (Fe^2+^; Grabb, Buchwald, Hansel, & Wankel, 2017; Wankel et al., 2017). Boreal forested catchments, typically on podzols, generally have a high iron export to surface water (Ekström et al., 2016; Kortelainen et al., 2006) and thus are likely to offer conditions suitable for chemodenitrification and potentially high yields of N_2_O (Kulkarni, Yavitt, & Groffman, 2017).

Seasonality appeared to influence N_2_O concentrations in forest streams as N_2_O %sat measured in autumn was significantly higher than in the other seasons. A possible explanation is that when plants decay in autumn, more labile organic matter becomes available thus providing carbon and N to microbes that subsequently produce N_2_O. In agricultural streams, N_2_O emissions seemed slightly lower in summer and spring perhaps because N is rapidly processed and depleted by the vegetation and microbes during the growing season. Furthermore, N_2_O emissions from forest streams seemed to decrease with increasing stream order while N_2_O emissions were relatively constant in agricultural streams. A decrease in N_2_O emissions with increasing stream order is generally expected because most N transported to surface waters will primarily reach low‐order streams and is assumed to be rapidly processed before being transported downstream (Alexander, Boyer, Smith, Schwarz, & Moore, 2007; Marzadri, Dee, Tonina, Bellin, & Tank, 2017; Peterson et al., 2001). For example, a decrease in N_2_O emissions with increasing stream order was observed in a study from Minnesota, USA (Turner et al., 2015). However, we did not observe a similar pattern in our agricultural streams, perhaps because our data set comprised only low‐order streams from several regions, whereas Turner et al. (2015) investigated streams and rivers ranging in order from 1 to 10, located in the same region and with similar crop cover (mainly corn production).

It is unclear whether most N_2_O in streams is produced in upland or riparian soils before being transported to surface waters or whether it is produced in situ. Upland forest soils are generally believed to act as weak sources or sinks of atmospheric N_2_O, and production of N_2_O can proceed through both nitrification and denitrification (Laverman, Zoomer, & Verhoef, 2001; Peichl, Arain, Ullah, & Moore, 2009; Skiba, Pitcairn, Sheppard, Kennedy, & Fowler, 2005). Increased N_2_O production has been observed after both increasing moisture content and increased N load (Sitaula & Bakken, 1993; Ullah, Frasier, King, Picotte‐Anderson, & Moore, 2008), and this N_2_O could then be transferred from soils to streams. Additionally, the role of the riparian zone as source of N_2_O production needs to be clarified, considering the strong controls that it exerts on a wide range of biogeochemical processes in forested and agricultural catchments (Blackburn, Ledesma, Näsholm, Laudon, & Sponseller, 2017; Ledesma et al., 2018; Ranalli & Macalady, 2010). The proportion of wetlands in the catchment also strongly alters TN, DOC and iron dynamics in headwater forest streams (Löfgren, Fröberg, Yu, Nisell, & Ranneby, 2014; Sponseller et al., 2018) and thus can affect N_2_O production processes. In situ production of N_2_O in the hyporheic and benthic zones of the stream is suggested to be a major source of stream N_2_O (Marzadri et al., 2017). However, a study of 72 headwater streams determined that in‐stream denitrification contributed, on average, only 26% of the total N_2_O emissions (Beaulieu et al., 2011), whereas the contribution by other processes (e.g., nitrification or chemodenitrification) remains largely unknown.

Our estimate of the national emission of N_2_O from Swedish streams provides compelling evidence that streams should be considered as significant sources of N_2_O in global GHG inventories. In accordance with our second hypothesis, we highlight the importance of forest streams for N_2_O emissions as more than 80% of the Swedish stream emissions occurred in forest ecosystems. Obviously, this large share is partly explained by the fact that forest streams constitute 74% of the total surface area of the stream network in Sweden, while agricultural streams account for 21%. The total stream emission of ~1,780 × 10^6^ g N_2_O‐N/year (corresponding to ~830 × 10^9^ g _2_‐eq) would represent 25% of the total N_2_O emission from the agricultural sector in Sweden, which was estimated to be ~3.2 × 1,012 g CO_2_‐eq in 2015 (Swedish Environmental Protection Agency, 2016).

The national N_2_O emission from Swedish streams estimate should be interpreted with caution due to potentially large uncertainties. For example, more measurements of the gas transfer velocity *k* or measurements of actual N_2_O emissions are needed to generate more robust estimates of stream N_2_O emissions at a national scale. The absence of a clear difference between area‐based N_2_O emissions from forest and agricultural streams might be partly due to the relatively low TN concentration measured at UPP1‐2 compared with those of other agricultural catchments in Sweden (Kyllmar et al., 2014). Furthermore, spring and summer N_2_O values constituted 72% of the whole data set for agricultural sites and this might have biased our yearly estimates considering that spring and summer N_2_O concentrations seemed slightly lower than during the other seasons. The role of seasonality on N_2_O emissions is unclear as some studies have found higher N_2_O emissions in summer and autumn (Tian, Zhu, & Akiyama, 2017), while others found lower emissions in summer (Beaulieu et al., 2008) or no seasonal trend (Baulch, Schiff, Maranger, & Dillon, 2011). N_2_O emissions from agricultural streams might also be more variable spatially and temporally compared to forest streams because of artificial drainage of the soil and fertilization practices. Hence hot spots (e.g., drain pipes outlets) and hot moments of N_2_O emissions might have been missed, especially considering that N_2_O transported in stream water can be rapidly outgassed to the atmosphere within a few hundred meters of stream length (Reay et al., 2003). Taken together, our national estimate of stream N_2_O emission might underestimate the contribution from agricultural streams. Still, in spite of these uncertainties, the results point to a substantial contribution of low‐order streams, including forest streams, to the total emissions of N_2_O. Hence, when compared with the estimate of CO_2_ and CH_4_ emission (in CO_2_‐eq) from Swedish headwater streams, N_2_O emission (in CO_2_‐eq) would represent ~7% of the total GHG stream emission, which is as much as the diffusive CH_4_ emission (Wallin et al., 2018). Our results provide new evidence for the importance of forest and agricultural streams as substantial sources of N_2_O to the atmosphere. In particular, N_2_O emissions from forest streams should be taken into account in GHG inventories, considering that boreal coniferous forests are among the largest biomes on Earth with ~8 M km^2^, that is, 30% of the global forest area (Gauthier, Bernier, Kuuluvainen, Shvidenko, & Schepaschenko, 2015; Hansen et al., 2013).

This study suggests that even relatively low N levels processed and leached to surface waters via acidic soils in forested catchments can yield significant amounts of N_2_O emitted to the atmosphere. Consequently, N deposition and fertilization of forest soils might lead to higher N_2_O emissions than anticipated if N is leached to surface water. Atmospheric N deposition reaches about 7 kg N/ha year in southern Sweden, 3.9 kg N/ha year in central Sweden and 1.2 kg N/ha year in northern Sweden (Lucas, Sponseller, & Laudon, 2013). In addition, ~33,200 ha of forest were fertilized in Sweden in 2015, mostly in the north (average fertilization was 150 kg N/ha year; Swedish Environmental Protection Agency, 2016). The Intergovernmental Panel on Climate Change (IPCC) includes the volatilization of N fertilizer applied onto agricultural or forest soils in the national inventories of anthropogenic emissions but does not consider whether the deposition will actually occur on agricultural or forest soils, which might have different N_2_O emission factors. According to IPCC guidelines, the ratio of volatilized N assumed to end up as N_2_O after redeposition is 0.01, that is, 1%. This ratio might be seriously underestimated when redeposition occurs on acidic soils such as the majority of coniferous forest soils in Sweden. Furthermore, there is a current debate whether the IPCC factor used to estimate riverine N_2_O emission (EF_5r_) should be adjusted. On one hand, several studies have suggested that the IPCC factor EF_5r_ is underestimated (e.g., Beaulieu et al., 2011; Turner et al., 2015). On the other hand, a recent paper, using a modeling approach, concluded that N_2_O emissions from inland waters might be overestimated by an order of magnitude (Maavara et al., 2019). However, a review concluded that the IPCC factor for N_2_O riverine emission was actually very similar to the factor calculated from a global data set of N_2_O stream emissions (Tian, Cai, & Akiyama, 2019). In our study, we showed that the ratio N_2_O:${\text{NO}}_{3}^{-}$ at the agricultural streams (0.0015) compared well with the EF_5r_ (0.0025) while the forest streams seemingly had a higher emission factor (0.011). Hence, there is still a great need to better constrain estimates of riverine N_2_O emissions, especially in forest streams.
