# Supplementary material for: Forest streams are important sources for nitrous oxide emissions
Source: Glob Chang Biol. 2019 Sep 25;26(2):629–41. doi: 10.1111/gcb.14812 (PMC7027446; doi:10.1111/gcb.14812)
Supplement: Supplementary file 1 [file GCB-26-629-s001.docx]

**Supporting information:**

**
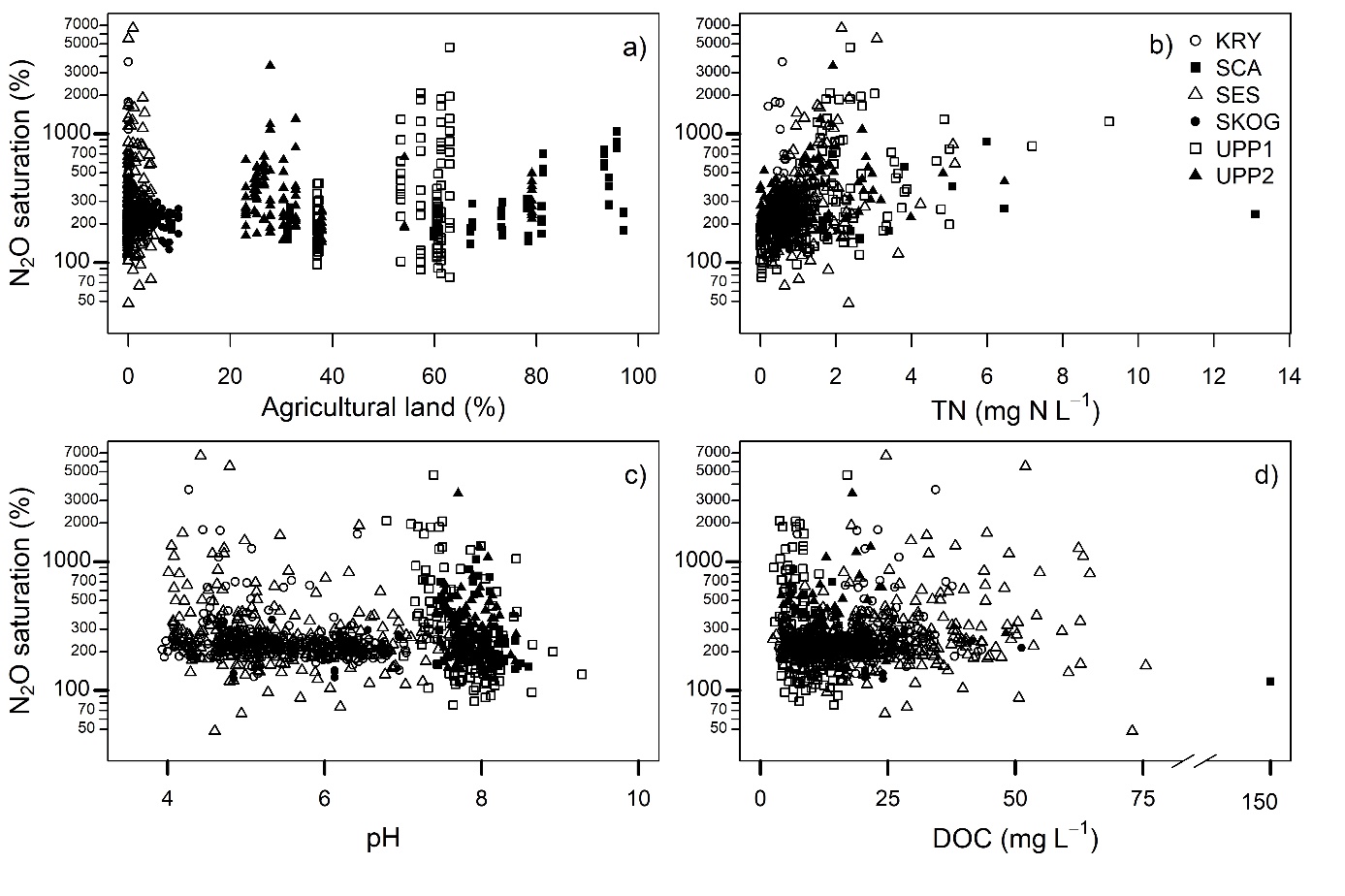
**


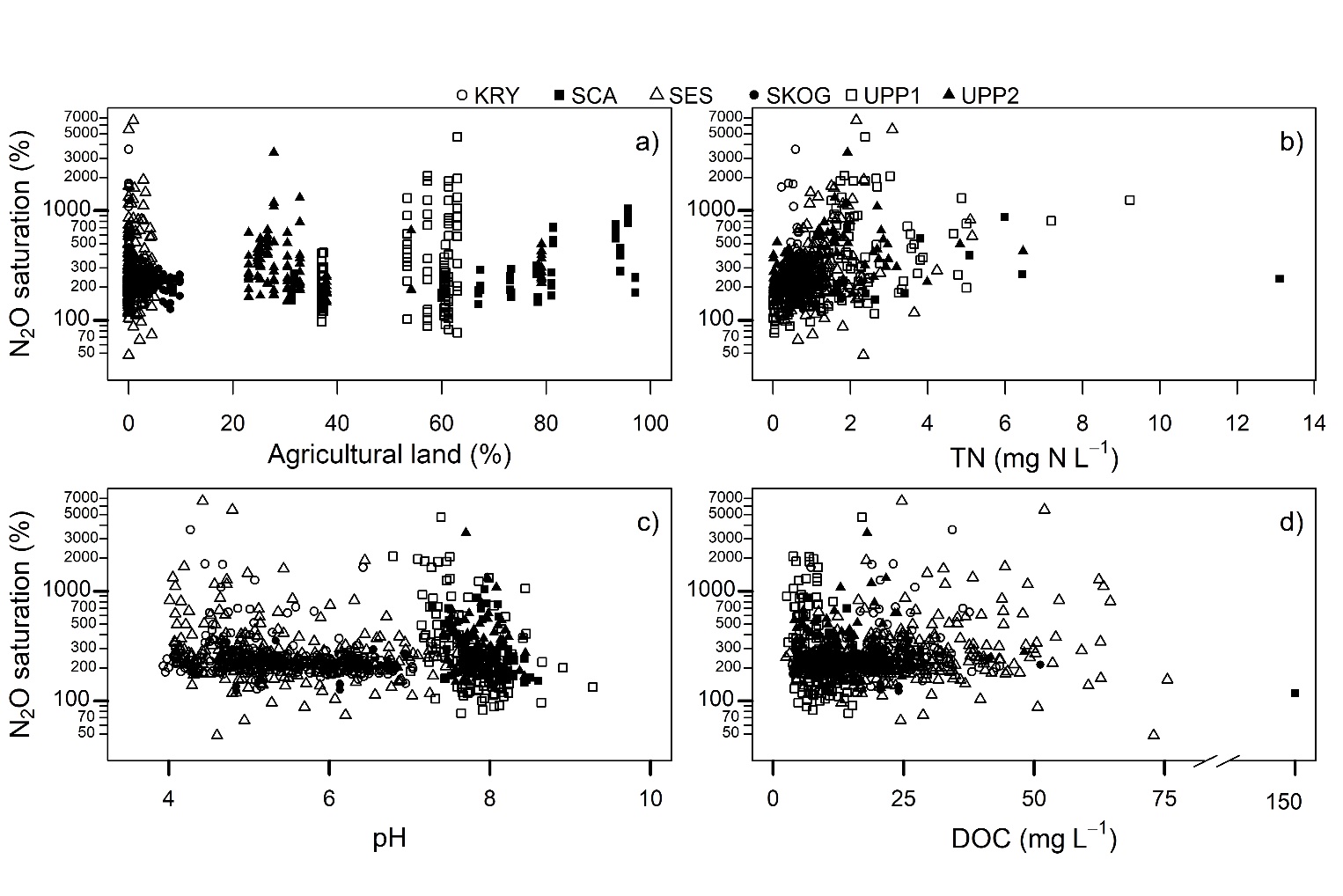
 Figure S1: Percent of N_2_O saturation in the stream against a) share of agricultural land within the catchment, b) TN concentration, c) pH, and d) DOC concentration in stream water.

| Linear mixed models | Response variable | Fixed effects | Random effects | Parameter estimates | Value | Std Error | 95% CI | *n* | DF | t-value | p-value |
| --- | --- | --- | --- | --- | --- | --- | --- | --- | --- | --- | --- |
| Model 1 | ln(N_2_O *%sat)* | Land use (i.e. forest or agriculture) | Regions | Intercept | 5.65 | 0.06 | 5.53–5.77 | 928 | 922 | 85.21 | **<0.001** |
|  |  |  |  | Forest | -0.15 | 0.08 | -0.38–0.08 | 928 | 922 | -1.84 | 0.14 |
| Model 2 | TN (mg N L^-1^) | Land use | Regions | Intercept | 1.49 | 0.22 | 1.06–1.92 | 636 | 629 | 6.83 | **<0.001** |
|  |  |  |  | Forest | -0.78 | 0.28 | -1.56–-0.01 | 636 | 629 | -2.79 | **0.049** |
| Model 3 | pH | Land use | Regions | Intercept | 7.87 | 0.04 | 7.78–7.96 | 952 | 946 | 175.70 | **<0.001** |
|  |  |  |  | Forest | -2.41 | 0.07 | -2.61–-2.20 | 952 | 946 | -32.80 | **<0.001** |
| Model 4 | DOC (mg L^-1^) | Land use | Regions | Intercept | 9.81 | 1.86 | 6.15–13.45 | 909 | 903 | 5.27 | **<0.001** |
|  |  |  |  | Forest | 13.15 | 2.68 | 5.73–20.58 | 909 | 903 | 4.91 | **0.008** |
| Model 5 | ln(N_2_O *%sat)* (Forest streams) | Season | Regions | Intercept | 5.91 | 0.06 | 5.79–6.04 | 630 | 624 | 91.60 | **<0.001** |
|  |  |  |  | Spring | -0.40 | 0.07 | -0.53–-0.27 | 630 | 624 | -6.04 | **<0.001** |
|  |  |  |  | Summer | -0.58 | 0.07 | -0.72–-0.45 | 630 | 624 | -8.32 | **<0.001** |
|  |  |  |  | Winter | -0.66 | 0.08 | -0.82–0.50 | 630 | 624 | -8.18 | **<0.001** |
| Model 6 | ln(N_2_O *%sat)* (Agricultural streams) | Season | Regions | Intercept | 5.80 | 0.12 | 5.56–6.04 | 298 | 292 | 48.00 | **<0.001** |
|  |  |  |  | Spring | -0.13 | 0.13 | -0.38–0.12 | 298 | 292 | -0.98 | 0.33 |
|  |  |  |  | Summer | -0.39 | 0.10 | -0.59–0.18 | 298 | 292 | -3.75 | **<0.001** |
|  |  |  |  | Winter | 0.44 | 0.18 | -0.09–0.79 | 298 | 292 | 2.44 | **0.015** |
| Model 7 | ln(N_2_O %sat) (Forest streams) | Stream order | Regions | Intercept | 5.50 | 0.04 | 5.52–5.57 | 608 | 602 | 143.83 | **<0.001** |
|  |  |  |  | Order 2 | -0.02 | 0.04 | -0.10–0.06 | 608 | 602 | -0.57 | 0.57 |
|  |  |  |  | Order 3 | -0.13 | 0.04 | -0.21–-0.04 | 608 | 602 | -3.02 | **0.003** |
|  |  |  |  | Order 4 | -0.18 | 0.05 | -0.28–0.39 | 608 | 602 | -3.24 | **0.001** |
| Model 8 | ln(N_2_O %sat) (Agricultural streams) | Stream order | Regions | Intercept | 5.65 | 0.08 | 5.48–5.81 | 298 | 291 | 67.12 | **<0.001** |
|  |  |  |  | Order 2 | -0.06 | 0.10 | -0.25–0.13 | 298 | 291 | -0.66 | 0.51 |
|  |  |  |  | Order 3 | -0.10 | 0.13 | -0.36–-0.16 | 298 | 291 | -0.78 | 0.44 |
|  |  |  |  | Order 4 | 0.38 | 0.14 | 0.09–0.66 | 298 | 291 | 2.62 | **0.009** |
|  |  |  |  | Order 5 | 0.20 | 0.15 | -0.10–0.50 | 298 | 291 | 1.28 | 0.20 |
| Model 9 | ln(N_2_O *%sat)* | ln(TN) + DOC + pH + %agricultural land + %wetland + Water temperature | Regions | See table 3 |  |  |  |  |  |  |  |
|  |  |  |  |  |  |  |  |  |  |  |  |

Table S1: Description and results of the linear mixed models. CI, Confidence Interval; DF, degree of freedom; *n*, number of observations. Significant results are shown in bold.

| Linear mixed models | Levels tested | Estimate | Std. Error | z value | Pr(>\|z\|) |
| --- | --- | --- | --- | --- | --- |
| Model 5 | Spring - Autumn | -0.40 | 0.07 | -6.06 | **<0.001** |
|  | Summer - Autumn | -0.58 | 0.07 | -8.35 | **<0.001** |
|  | Winter - Autumn | -0.66 | 0.08 | -8.20 | **<0.001** |
|  | Summer - Spring | -0.18 | 0.03 | -5.61 | **<0.001** |
|  | Winter - Spring | -0.26 | 0.05 | -5.00 | **<0.001** |
|  | Winter - Summer | -0.08 | 0.06 | -1.41 | 0.47 |
| Model 6 | Spring - Autumn | -0.13 | 0.13 | -0.99 | 0.75 |
|  | Summer - Autumn | -0.39 | 0.10 | -3.77 | **< 0.001** |
|  | Winter - Autumn | 0.44 | 0.18 | 2.46 | 0.06 |
|  | Summer - Spring | -0.26 | 0.12 | -2.22 | 0.11 |
|  | Winter - Spring | 0.57 | 0.18 | 3.16 | **0.008** |
|  | Winter - Summer | 0.83 | 0.17 | 4.85 | **< 0.001** |
| Model 7 | 2 - 1 | -0.02 | 0.04 | -0.57 | 0.94 |
|  | 3 - 1 | -0.13 | 0.04 | -3.03 | **0.012** |
|  | 4 - 1 | -0.18 | 0.05 | -3.26 | **0.006** |
|  | 3 - 2 | -0.11 | 0.05 | -2.21 | 0.12 |
|  | 4 - 2 | -0.16 | 0.06 | -2.61 | **0.043** |
|  | 4 - 3 | -0.05 | 0.06 | -0.79 | 0.86 |
| Model 8 | 2 - 1 | -0.06 | 0.10 | -0.66 | 0.96 |
|  | 3 - 1 | -0.10 | 0.13 | -0.79 | 0.93 |
|  | 4 - 1 | 0.38 | 0.14 | 2.64 | 0.06 |
|  | 5 - 1 | 0.20 | 0.15 | 1.29 | 0.68 |
|  | 3 - 2 | -0.04 | 0.11 | -0.35 | 1.00 |
|  | 4 - 2 | 0.44 | 0.13 | 3.51 | **0.004** |
|  | 5 - 2 | 0.26 | 0.14 | 1.90 | 0.30 |
|  | 4 - 3 | 0.48 | 0.15 | 3.12 | **0.015** |
|  | 5 - 3 | 0.30 | 0.16 | 1.84 | 0.34 |
|  | 5 - 4 | -0.18 | 0.17 | -1.03 | 0.84 |

Table S2: Comparison of the different levels of the mixed models using Tukey’s post-hoc test. Significant results are shown in bold. The levels are seasons for Model 5 and 6, and stream orders for model 7 and 8.
